# TSEDTA: a transformer-based neural network with SMILES transformer and ESM2 embeddings for drug-target binding affinity prediction

**DOI:** 10.1093/bioinformatics/btag298

**Published:** 2026-05-09

**Authors:** Xu Sun, Xiaoying Liu, Juanjuan Huang, Jiageng Wu, Yuchen Sun, Jiwei Jia

**Affiliations:** Department of Computational Mathematics, School of Mathematics, Jilin University, Changchun, 130012, China; Department of Computational Mathematics, School of Mathematics, Jilin University, Changchun, 130012, China; Department of Computational Mathematics, School of Mathematics, Jilin University, Changchun, 130012, China; Department of Laboratory Medicine, Zhengzhou Central Hospital Affiliated to Zhengzhou University, Zhengzhou, 450007, China; Department of Computational Mathematics, School of Mathematics, Jilin University, Changchun, 130012, China; School of Advanced Manufacturing and Robotics, Peking University, Beijing, 100871, China; Department of Computational Mathematics, School of Mathematics, Jilin University, Changchun, 130012, China; Department of Computational Mathematics, School of Mathematics, Jilin University, Changchun, 130012, China; AI for Science and Engineering Center, Shenzhen Loop Area Institute, Shenzhen, 518048, China

## Abstract

**Motivation:**

Drug-target binding affinity (DTA) prediction plays a vital role in drug repositioning. The emergence of large language models (LLMs) has introduced new perspectives for predicting DTA. Herein, we present TSEDTA, a Transformer-based neural network with SMILES Transformer and ESM2 embeddings for predicting DTA. It leverages pre-trained LLMs (SMILES Transformer and ESM2) to extract deep evolutionary representations from drug SMILES and protein sequences. The representations are directly fused with raw sequence embeddings and processed via dual Transformer encoders to capture complex local and global dependencies.

**Results:**

The experiments demonstrate that TSEDTA outperforms ten advanced models on the Davis and KIBA datasets, and seven on the BindingDB dataset. Ablation studies show that incorporating LLM embeddings significantly improves the performance of TSEDTA. Furthermore, a practical case study demonstrates its real-world applicability. Ultimately, TSEDTA provides a highly accurate, robust tool for DTA prediction, offering new insights into the application of LLMs for DTA tasks.

**Availability:**

The source code and data are available at: https://github.com/SunXu24Math/TSEDTA. The version of record is archived in Zenodo with the DOI: 10.5281/zenodo.19103249.

## 1 Introduction

Drug repositioning, in which existing drugs are used to treat new diseases, is an effective strategy for accelerating drug development and lowering costs (Ashburn and Thor 2004, Pushpakom *et al.* 2019). The prediction of drug–target binding affinity (DTA) plays a vital role in this process (Sydow *et al.* 2019). Traditional experimental methods for predicting DTA require considerable time and manual workload (Rognan 2010, Yang *et al.* 2021). In contrast, computational methods are more efficient and easier to implement, thereby significantly accelerating drug repositioning. Thus, they serve as a direct and essential complement to experimental methods for DTA prediction (Rognan 2010, Sydow *et al.* 2019, Lin *et al.* 2020b, Yang *et al.* 2021).

Numerous computational approaches have been developed to predict DTA. KronRLS constructs a kernel function based on similarity matrices and applies the Kronecker RLS for DTA prediction by minimizing the objective function (Pahikkala *et al.* 2015). SimBoost utilizes drug SMILES, target sequence similarity information, and matrix factorization results to derive features, subsequently employing a gradient boosting machine to capture their nonlinear associations with binding affinity (He *et al.* 2017). SimCNN-DTA employs a two-dimensional convolutional neural network (CNN) using the outer product of the column vectors from drug/target Tanimoto similarity matrices and Smith-Waterman similarity matrices for predicting DTA (Shim *et al.* 2021). DeepDTA represents drugs and proteins using integer or label encoding and applies separate CNNs to extract features for DTA prediction (Öztürk *et al.* 2018). DeepGS employs advanced embedding techniques to convert sequences into distributed representations and builds a deep learning (DL) network to predict DTA (Lin et al. 2020a). GANsDTA adopts generative adversarial networks (GANs) to learn representations of protein sequences and the drug SMILES, followed by affinity estimation using a CNN (Zhao *et al.* 2019).

More recently, attention-based and advanced generative architectures have emerged to capture complex molecular interactions. For instance, methods leveraging structural representations, such as GraphDTA (Nguyen *et al.* 2021) and Affinity2Vec (Thafar *et al.* 2022), utilize graph mining and GNNs to effectively model the topological information of drugs. Similarly, sequence-based deep learning models have increasingly adopted attention mechanisms (Attention DTA (Zhao *et al.* 2023); MATT_DTI (Zeng *et al.* 2021)) to pinpoint critical binding sites between drugs and targets. Furthermore, the introduction of end-to-end Transformer architectures (DTITR (Monteiro *et al.* 2022)) and multi-scale interactive learning paradigms (MDCT-DTA (Zhu *et al.* 2024)) have pushed the boundaries of DTA prediction. Other notable recent works include CPInformer, which incorporates compound structure graphs and functional class fingerprints, fuses local and global protein features through densely connected layers, and applies the ProbSparse self-attention mechanism to reduce redundant information and improve DTA prediction (Hua *et al.* 2023). TransVAE-DTA uses a variational autoencoder for predicting the DTA (Zhou *et al.* 2024).

However, while these advanced methods have improved prediction accuracy, they still face significant limitations in feature representation depth and generalization capability. Specifically, unlike methods that depend on complex graph topologies (Nguyen *et al.* 2021, Thafar *et al.* 2022, Hua *et al.* 2023), or interactive diffusion processes (Zhu *et al.* 2024) that may miss deep semantic biochemical rules, there is a critical need for a precise, generalizable and information-preserving DTA prediction method. This requirement motivated the design of our proposed approach. The recent emergence of large language models (LLMs) has offered promising solutions to these challenges. For protein molecules, Evolutionary Scale Modeling (ESM2) captures hidden structural and functional features from protein sequences, successfully learning latent evolutionary characteristics (Rives *et al.* 2021). For drug molecules, the SMILES Transformer encodes SMILES representations using the Transformer architecture to learn continuous data-driven molecular fingerprints that grasp the underlying semantics (Honda *et al.* 2019).

In this article, we propose a Transformer-based neural network with LLM embeddings for predicting DTA (named TSEDTA). It combines pretrained LLMs for feature extraction with Transformer architectures for feature learning. TSEDTA is composed of three key modules: LLM-Fusion block, Dual-Trans block, and DTA prediction. In the LLM-Fusion block, we utilize SMILES Transformer for drugs and ESM2 for targets to obtain contextual embeddings. These embeddings are mapped to a unified projection dimension and fused with the original sequence embeddings and positional encodings. We then applied two independent Transformer encoders to the fused drug and protein embeddings in the Dual-Trans Block. Finally, the encoded features are passed through sequential dense layers to learn the drug-target interaction and predict the final DTA score. We comprehensively evaluated TSEDTA across four benchmark datasets. The results show that TSEDTA outperformed nine advanced models. Moreover, ablation studies validate the contribution of each pre-trained LLM to the predictive performance. Overall, TSEDTA demonstrates strong potential as a powerful and reliable tool for predicting DTA.

## 2 Methods

### 2.1 Overview of TSEDTA

**Figure 1:**
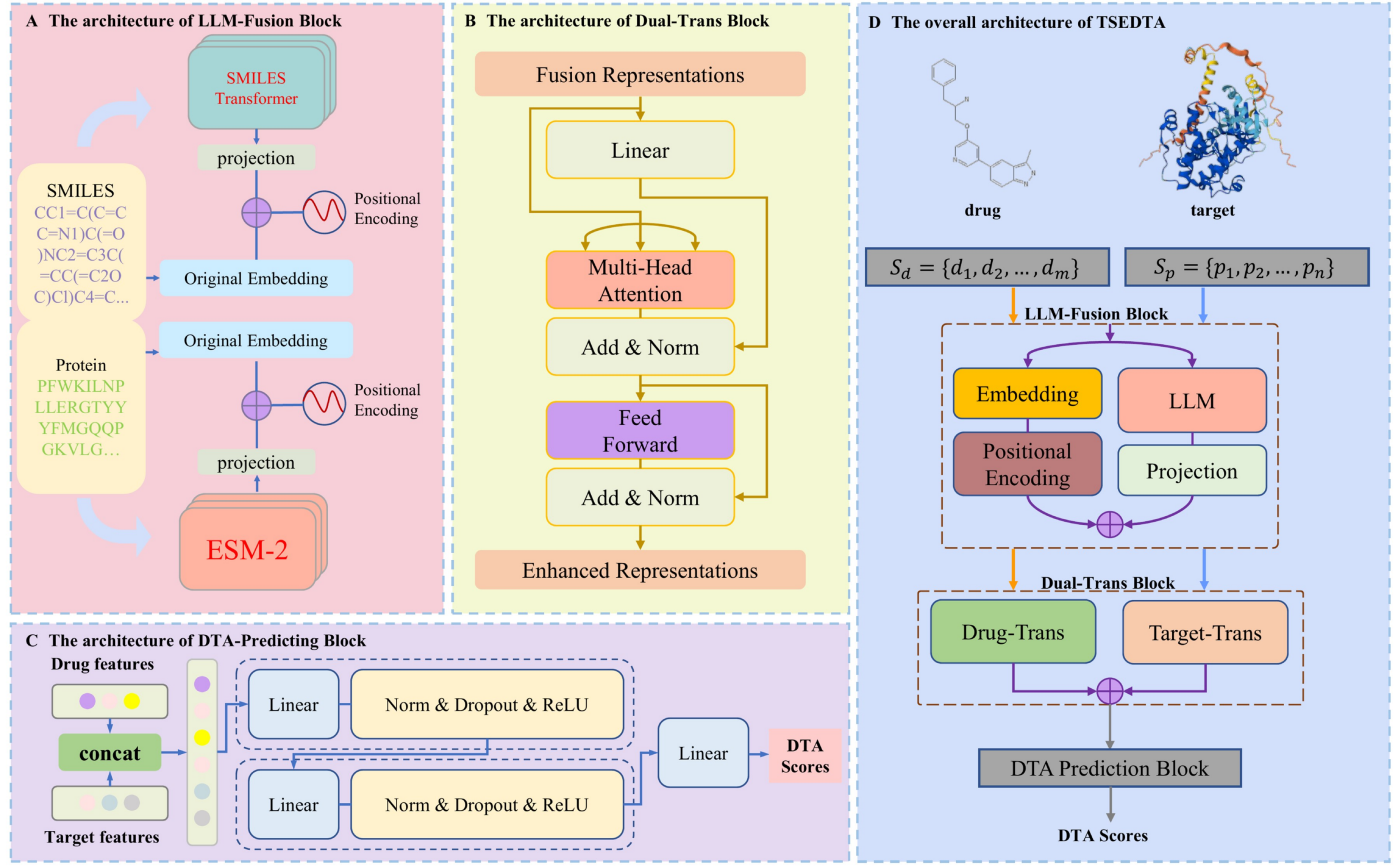
An overview of TSEDTA architecture for DTA prediction. (A–C) The architecture of LLM-Fusion block (A), Dual-Trans block (B) and DTA prediction block (C). (D) The overall architecture of TSEDTA.

TSEDTA is a novel framework for DTA prediction, integrating pretrained LLMs and Transformer encoders to learn features of drug SMILES and protein sequences. It consists of three key modules: LLM-Fusion block for preliminary feature extraction, Dual-Trans block for feature enhancement, and DTA prediction block. Figure 1 presents the overall framework of TSEDTA. First, drug SMILES and protein sequences are put into LLM-Fusion block, where embeddings are extracted using two pretrained LLMs. The SMILES Transformer and ESM2 are employed as frozen feature extractors; their parameters remain fixed during the training of TSEDTA to preserve generalized biochemical knowledge and reduce computational costs. The embeddings are then concatenated with the raw sequence embeddings to preserve original information. Next, Dual-Trans block applies dual Transformer encoders to enhance the representations of drugs and proteins. Finally, DTA prediction block integrates the concatenated features and predicts DTA scores.

### 2.2 LLM-Fusion block

This block generates comprehensive representations of drug SMILES *S_d_* and protein sequences *S_p_* by combining the original sequence with embeddings produced by pre-trained LLMs.

We employ the pre-trained SMILES Transformer (S-T) and ESM2 to obtain contextual embeddings.


(1) $$L_{d}=S–T(S_{d})\in R^{m\times d_{1}},\;L_{p}=\text{ESM}2(S_{p})\in R^{n\times d_{2}}$$

*m*, *n* denote the lengths of drug SMILES and target sequences, and d_1_, d_2_ denote the embedding dimensions of SMILES Transformer and ESM2.

To project both embeddings into the same feature space and fuse with original sequence features, we introduce a projection layer applied to the LLM embeddings to map them into a pre-defined projection dimension d.


(2) $$\hat{E_{d}}=F(L_{d})\in R^{m\times d},\;\hat{E_{p}}=F(L_{p})\in R^{n\times d}$$

Where Ȇ_d_, Ȇ_p_ denote the dimension-aligned representations of drugs and targets obtained through the learnable projection function ℱ.

#### 2.1.1 Drug representation via SMILES transformer

SMILES Transformer (S-T) is a pretrained Transformer-based architecture, commonly used for molecular representation learning and property prediction tasks (Honda *et al.* 2019). In contrast to traditional Recurrent Neural Networks (RNNs), the Transformer architecture does not rely on recurrent connections, thus offering enhanced stability, faster convergence and superior performance in modeling long sequences and complex dependencies (Vaswani *et al.* 2017).

All atomic symbols, special characters (e.g., parentheses, bond symbols) and two-character elements (e.g., “Cl” and “Br”) are identified from the CHEMBL database to form the token set T:


(3) $$T=\{t_{1},t_{2},\ldots ,t_{\left|T\right|}\}$$

Where *t_i_* denotes the *i*-th identified token (Zdrazil *et al.* 2024).

Based on this token set, a vocabulary mapping *V_d_* is defined to assign a unique integer index to each token.


(4) $$V_{d}:T\to \{1,2,\ldots ,|T|\},\;V_{d}(t_{i})=i$$

The drug SMILES *S_d_* is tokenized into a sequence of symbols using a pre-defined regular expression pattern. For example,


$$S_{d}=\text{CN}1C\ldots )\text{Br})F)\text{OC}\Rightarrow \{C,\;N\;,\;1,\;C,\ldots ,),\mathrm{Br},),F,),O,\;C\}$$

Each token in the sequence is then converted into its corresponding index based on the vocabulary *V_d_*.

S-T is pretrained on large-scale SMILES corpora. It learns molecular representations by reconstructing the input sequences using an encoder-decoder architecture. The input drug SMILES *S_d_* is first encoded by a Transformer encoder (T-E) and then reconstructed by a Transformer decoder (T-D) to produce *S^_d_* as follows:


(5) $$\hat{S_{d}}=T–D(T–E(S_{d}))$$

The output of the Transformer encoder $T–E(S_{d})$ serves as the continuous molecular representation, referred to as the S-T fingerprint.

#### 2.2.2 Target represention via ESM2

ESM2 is a protein LLM built upon the RoBERTa (Liu *et al.* 2019) architecture, which is based on the Transformer framework. It is pre-trained on the Uniref50 protein corpus, allowing it to learn both structural and functional features directly from amino acid sequences (Rives *et al.* 2021).

For a protein sequence *S_p_*, we represent it as an embedding vector *L_p_* using ESM2. To reduce computational complexity, sequences exceeding a predefined maximum length are truncated before entering ESM2:


(6) $$S_{p}=(p_{1},p_{2},\ldots ,p_{n})\Rightarrow \tilde{S_{p}}=(p_{1},p_{2},\ldots ,p_{\mathrm{maxlen}})$$

Where *S^∼^_p_* denotes the actual input sequence to ESM2.

We generate pretrained 1280-dimensional embeddings via the output of the 33rd layer of ESM2, formulated as:


(7) $$L_{p}={\text{ESM}2}_{\text{Layer}=33}(\tilde{S_{p}})\in R^{n\times 1280}$$

Meanwhile, to ensure consistency in tokenization throughout the entire model, we used the same amino acid vocabulary A in ESM2 for subsequent encoding.


(8) $$p_{i}\in A,\;V_{p}:A\to \{1,2,\ldots ,|A|\},\;V_{p}(p_{i})=i$$

#### 2.2.3 Embedding concatenation

In the LLM-Fusion block, the original drug and target sequences are first processed by LLMs to obtain rich contextual features Ȇ_d_ and Ȇ_p_, as described in Equation (2). To avoid losing the original sequence information, we concatenate the embeddings generated by the two LLMs with the raw sequence embeddings. In addition, positional encodings are incorporated into each sequence to effectively capture the positional relationships among tokens.

The drug SMILES *S_d_* = (*d*_1_, *d*_2_, …, *d_m_*) and target sequence *S_p_* = (*p*_1_, *p*_2_, *p_n_*) are first tokenized and converted into integer indices based on the predefined vocabulary. These indices are then mapped to high-dimensional vectors through an embedding layer.


(9) $$E_{d}=\mathrm{Embed}(S_{d})\in R^{m\times d},\;E_{p}=\mathrm{Embed}(S_{p})\in R^{n\times d}$$

Where Embed (·) denotes the embedding function, and d is the embedding dimension of whole model.

To incorporate positional information, sinusoidal position encodings PE_d_∈R^m×d^ and PE_p_∈R^n×d^ are added to the embeddings. The positional encoding at each position pos and dimension j is defined as Eq. S1.

Finally, the fused representations for drug SMILES and target sequences are computed as:


(10) $$R_{d}=E_{d}+\hat{E_{d}}+PE_{d},\;R_{p}=E_{p}+\hat{E_{p}}+PE_{p}$$

### 2.3 Dual-Trans block

In the Dual-Trans block, we further enhance both drug and target features, R_d_∈R^m×d^ and R_p_∈R^n×d^, using dual Transformer encoders. They pass through separate Transformer encoders as follows:


(11) $$H_{d}=\mathrm{Trans}(R_{d})\in R^{m\times d},\;H_{p}=\mathrm{Trans}(R_{p})\in R^{n\times d}$$

Where *H_d_* and *H_p_* are the enhanced features of the drug SMILES and target sequences.

The Transformer encoder, Trans (·), captures internal dependencies of input features mainly through self-attention block. Multi-head self-attention mechanism captures multiple types of dependencies in parallel. The details of the multi-head self-attention mechanism, Feed-Forward Network (FFN), and the normalization layers are provided in Eqs. S2–S6 in the Supplementary Information, available as supplementary data at *Bioinformatics* online.

After feature enhancement by the dual Transformer encoders, an average pooling is applied to H_d_ and H_p_ to obtain fixed-size global representations from variable-length sequences:


(12) $$r_{d}=\frac{1}{m}\sum_{i=1}^{m}H_{d}^{\left(i\right)},\;r_{p}=\frac{1}{n}\sum_{j=1}^{n}H_{p}^{\left(j\right)},\;r_{d},r_{p}\in R^{d}$$

Where *H_d_*^(i)^ and *H_p_*^(j)^ denote the *i-*th and *j*-th hidden vectors in the respective sequences.

The final unified representation is obtained by concatenating the two vectors, fusing features from both the drug and the target.


(13) $$r=[r_{d}|r_{p}]\in R^{2d}$$

### 2.4 DTA prediction

The drug and target representations obtained from the previous blocks are encoded as d-dimensional vectors. Their concatenation forms a 2D-dimensional input to this block. We first employ a multi-layer perceptron (MLP) composed of two sequential dense layers to project it into a higher-dimensional latent space. Each dense layer is composed of a linear transformation, layer normalization, dropout, and ReLU activation, which can be represented as:


(14) h=ReLU(Dropout(LayerNorm(Linear(r))))

Finally, a linear layer maps the drug-target features to a DTA score, represented as:


(15) $$\hat{y}=w^{⊤}h+b$$

Where *h* is the output of the last hidden layer, and ŷ∈R represents the predicted DTA score.

### 2.5 Datasets

We trained and evaluated TSEDTA on the Davis (Davis *et al.* 2011), KIBA (Tang *et al.* 2014), Metz (Metz *et al.* 2011) and BindingDB (Gilson *et al.* 2016) datasets. The Davis dataset consists of 68 drugs, 442 proteins, and 30 056 interaction pairs. The affinities are quantified by the dissociation constant *K_d_*. Because *K_d_* values are inconvenient to calculate, they are commonly transformed into pK_d_ values as follows:


(16) $$pK_{d}=-\log_{10}\left(\frac{K_{d}}{10^{9}}\right)$$

pK_d_ values range from 5.0 to 10.8, and higher values indicate stronger binding affinities. The KIBA dataset contains 2,111 drugs and 229 proteins, and 118 254 interaction pairs. The affinities are estimated by KIBA scores, which range from 0 to 17.2. The Metz dataset consists of 170 drugs, 1423 proteins, and 35 259 interaction pairs. The BindingDB dataset consists of 9864 drugs, 1088 proteins, and 42 201 interaction pairs. The pK_i_ values range from 4.0 to 11.1 in the Metz dataset, and 2.0 to 14.0 in the BindingDB dataset. A summary of these four datasets is provided in Table 1, available as supplementary data at *Bioinformatics* online.

**Table 1:** Parameters for four benchmark datasets.

Parameters	Davis	KIBA	Metz	BindingDB
Max Length of Drugs	85	100	80	100
Max Length of Proteins	1200	1000	1000	1000
Batch Size	32	32	64	32
Gradient Accumulation Steps	8	32	8	8
Initial Learning Rate	0.001	0.001	0.001	0.001
Dropout	0.1	0.1	0.3	0.1
Number of Epochs (Max)	600	600	600	600
Model Dimension	128	128	128	128
Feed-Forward Dimension	512	512	512	512
Count of Transformer Layers	1	1	1	1
Count of Attention Heads	4	4	4	4
SMILES Vocabulary Size	45	45	45	45
S-T Embedding Size	256	256	256	256
Proteins Vocabulary Size	33	33	33	33
ESM2 Embedding Size	1280	1280	1280	1280

### 2.6 Metrics

We adopted three widely used evaluation metrics (Concordance Index (CI), Mean Squared Error (MSE), and r_m_^2^) to evaluate the performance of TSEDTA.

CI measures the probability that the predicted binding affinity values preserve the correct rank order of the true values (Gönen and Heller 2005). It is defined as:


(17) $$\text{CI}=\frac{1}{Z}\sum_{y_{i}>y_{j}}h(p_{i}-p_{j})$$

Where *y_i_* and *y_j_* are the actual affinity values, *p_i_* and *p_j_* are the corresponding predicted values, and *Z* represents the number of comparable pairs satisfying *y_i_*>*y_j_*. The function *h*(*x*) is the Heaviside step function.

MSE measures the accuracy of the predicted DTA. The calculation formula of MSE is


(18) $$\text{MSE}=\frac{1}{n}\sum_{i=1}^{n}{\left(p_{i}-y_{i}\right)}^{2}$$

Where *n* is the total number of samples, *p_i_* is the predicted binding affinity, and *y_i_* is corresponding true value.

*r_m_* ^2^ evaluates the external predictive ability of the regression model (Roy *et al.* 2013).


(19) $$r_{m}^{2}=r^{2}(1-\sqrt{r^{2}-r_{0}^{2}})$$

Where *r*^2^ is the squared Pearson correlation coefficient between the predicted and true values, and $r_{0}^{2}$ is the squared correlation coefficient when the regression line is forced to pass through the origin.

### 2.7 Experimental settings

The four benchmark datasets were randomly divided into training and test subsets at a ratio of 5:1. To avoid data leakage, the test set was kept strictly isolated throughout training and parameter tuning, serving solely for the final evaluation. In addition, we conducted 5-fold cross-validation on the training set. During each fold, one part was used as the validation set and the other four were used for model training. Parameter tuning was performed based on the validation performance across the folds. The final evaluation was conducted on an independent test set using the best-performing model for cross validation. We selected the Adam optimizer for training because it is well-suited for tasks involving large-scale datasets. The MSE is used as the objective loss function. All other parameters are listed in Table 1.

Moreover, we employed an early stopping strategy and a learning rate scheduling mechanism during training. Specifically, the training was stopped early if the best metrics on the validation set plateaued for 30 consecutive epochs. And the learning rate scheduler automatically tuned the learning rate throughout training.

## 3 Results

### 3.1 Training process

**Figure 2:**
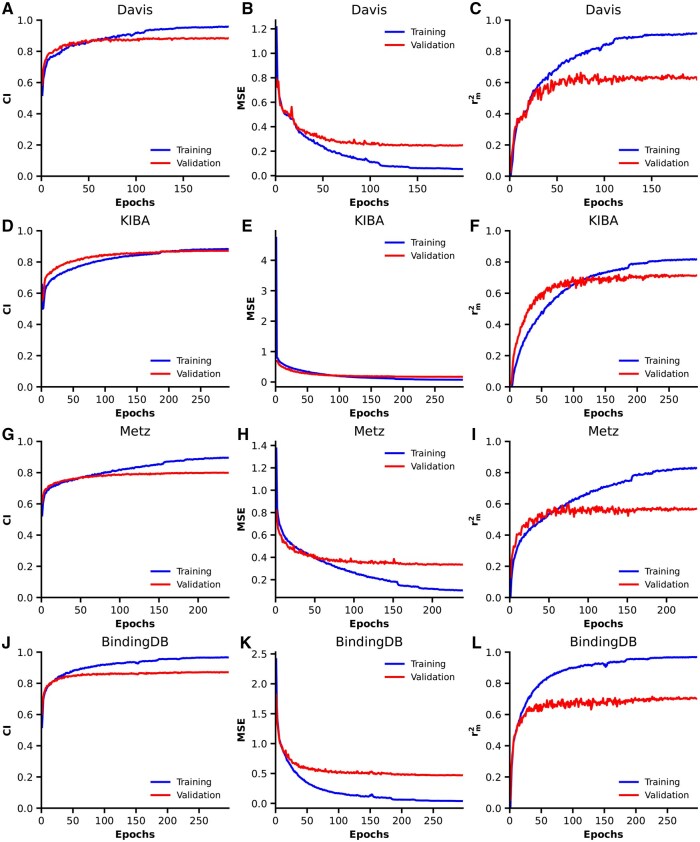
Training curves of TSEDTA on four benchmark datasets: Davis, KIBA, Metz, and BindingDB. (A–C) Results on the Davis dataset, including CI (A), MSE (B), and r_m_^2^ (C). (D–F) Results on the KIBA dataset. (G–I) Results on the Metz dataset. (J–L) Results on the BindingDB dataset.

Figure 2 shows the training process for CI, MSE, and *r_m_*^2^ across the four datasets. At the beginning of training, the MSE loss decreased rapidly, and the CI and *r_m_*^2^ values increased quickly. As training progressed, the improvements in all three metrics gradually decreased. During the first 40 epochs of the Davis dataset, the validation CI was higher than the training CI, whereas the MSE and r_m_^2^ values alternated in magnitude. For the KIBA dataset, the validation CI, MSE, and $r_{m}^{2}$ consistently outperformed the corresponding training metrics during the first 100 epochs. A similar trend was observed for the Metz dataset; during the initial 40 epoch, the validation metrics were slightly better than the training metrics. As the training proceeded, the training CI and *r_m_*^2^ gradually surpassed the validation values, while the training MSE decreased more rapidly than the validation MSE. For the BindingDB dataset, the MSE decreased sharply at the beginning of training, while CI and *r_m_*^2^ increased rapidly. During the early epochs, the validation metrics were close to the training metrics, but as training progressed, the training performance gradually exceeded the validation performance. In the later stages of training across all datasets, the metrics of both the training and validation sets gradually stabilized, indicating that the model approached convergence. Subsequent divergence between training and validation performance indicated the onset of overfitting, prompting the use of early stopping to select the optimal model.

### 3.2 Performance evaluation

We evaluated TSEDTA against representative state-of-the-art baseline models across four benchmark datasets. The detailed comparison results are summarized in Tables 2–4 and Table 2, available as supplementary data at *Bioinformatics* online.

**Table 2:** Performance comparison between TSEDTA and other models on Davis dataset.

Method	CI	MSE	$r_{m}^{2}$
DeepDTA (Öztürk *et al.* 2018)	0.878	0.261	0.63
GANsDTA (Zhao *et al.* 2019)	0.88	0.271	0.653
SimCNN-DTA (Shim *et al.* 2021)	0.852	0.319	0.595
MATT_DTI (Zeng *et al.* 2021)	0.884	0.254	0.649
CPInformer (Hua *et al.* 2023)	0.874	0.277	0.621
Affinity2Vec (Thafar *et al.* 2022)	0.887	0.24	**0.693**
TransVAE-DTA (Zhou *et al.* 2024)	0.8696	0.3329	0.5713
DMIL-PPDTA (Wang *et al.* 2022)	0.88	0.223	0.642
TF-DTA (Li *et al.* 2023)	0.886	0.231	0.67
GDilatedDTA (Zhang *et al.* 2024)	0.885	0.237	0.686
**TSEDTA**	**0.8891**	**0.2154**	0.6735

*Note*. Bold values indicate the best results in each column.

**Table 3:** Performance comparison between TSEDTA and other models on KIBA dataset.

Method	CI	MSE	$r_{m}^{2}$
DeepDTA (Öztürk *et al.* 2018)	0.863	0.194	0.673
DeepCPI (Wan *et al.* 2019)	0.852	0.211	0.657
GANsDTA (Zhao *et al.* 2019)	0.866	0.224	0.675
DeepGS (Lin et al. 2020a)	0.86	0.193	0.684
SimCNN-DTA (Shim *et al.* 2021)	0.821	0.274	0.573
TransVAE-DTA (Zhou *et al.* 2024)	0.8221	0.2536	0.6329
GraphDTA (Nguyen *et al.* 2021)	0.808	0.251	0.631
DeepGLSTM (Mukherjee *et al.* 2022)	0.855	0.185	0.705
CPInformer (Hua *et al.* 2023)	0.867	0.183	0.678
MambaTransDTA (Lou *et al.* 2026)	0.871	0.173	0.722
**TSEDTA**	**0.8745**	**0.1688**	**0.7308**

*Note*. Bold values indicate the best results in each column.

**Table 4:** Performance comparison between TSEDTA and other models on BindingDB dataset.

Method	CI	MSE	$r_{m}^{2}$
KronRLS (Pahikkala *et al.* 2015)	0.815	0.939	–
DeepDTA (Öztürk *et al.* 2018)	0.826	0.703	0.669
DeepCDA (Abbasi *et al.* 2020)	0.822	0.808	0.631
GraphDTA (Nguyen *et al.* 2021)	0.855	0.593	0.682
DoubleSG-DTA (Qian *et al.* 2023)	0.862	0.533	**0.726**
ELECTRA-DTA (Wang *et al.* 2022)	0.837	0.65	0.67
MambaTransDTA (Lou *et al.* 2026)	0.817	0.715	0.637
**TSEDTA**	**0.8698**	**0.5018**	0.7018

*Note*. Bold values indicate the best results in each column.

Davis Dataset (Table 2): Compared with methods illustrated in Table 2, TSEDTA achieved the best performance in CI (0.8891) and MSE (0.2154). Our model improved the CI by approximately 0.24% and decreased the MSE by 3.41%. The *r_m_*^2^ of TSEDTA (0.6735) was highly competitive, ranking just behind Affinity2Vec (0.693) and GDilatedDTA (0.686) with marginal differences of 0.0195 and 0.0125, respectively.

KIBA Dataset (Table 3): TSEDTA consistently outperformed all other advanced models across all three metrics, achieving the best CI (0.8745), MSE (0.1688), and *r_m_*^2^ (0.7308). Specifically, compared to the strongest competitor, MambaTransDTA, TSEDTA yielded a 0.40% improvement in CI, a 2.43% reduction in MSE, and a 1.22% improvement in *r_m_*^2^.

Metz Dataset (Table 2, available as supplementary data at *Bioinformatics* online): We also evaluated our model on the Metz dataset as detailed in Table 2, available as supplementary data at *Bioinformatics* online. While TSEDTA achieved CI, MSE, and *r_m_*^2^ values of 0.7955, 0.3346, and 0.5710 respectively, it did not outperform the top baseline methods on this specific dataset. A detailed discussion regarding this performance limitation and the dataset characteristics is provided in the Discussion section.

BindingDB Dataset (Table 4): TSEDTA achieved the highest performance in CI (0.8698) and MSE (0.5018) among all compared methods. Furthermore, its *r_m_*^2^ (0.7018) was the second-best, only 0.0242 lower than that of DoubleSG-DTA.

Overall, the above results show that TSEDTA achieved superior performance on multiple benchmark datasets.

**Figure 3:**
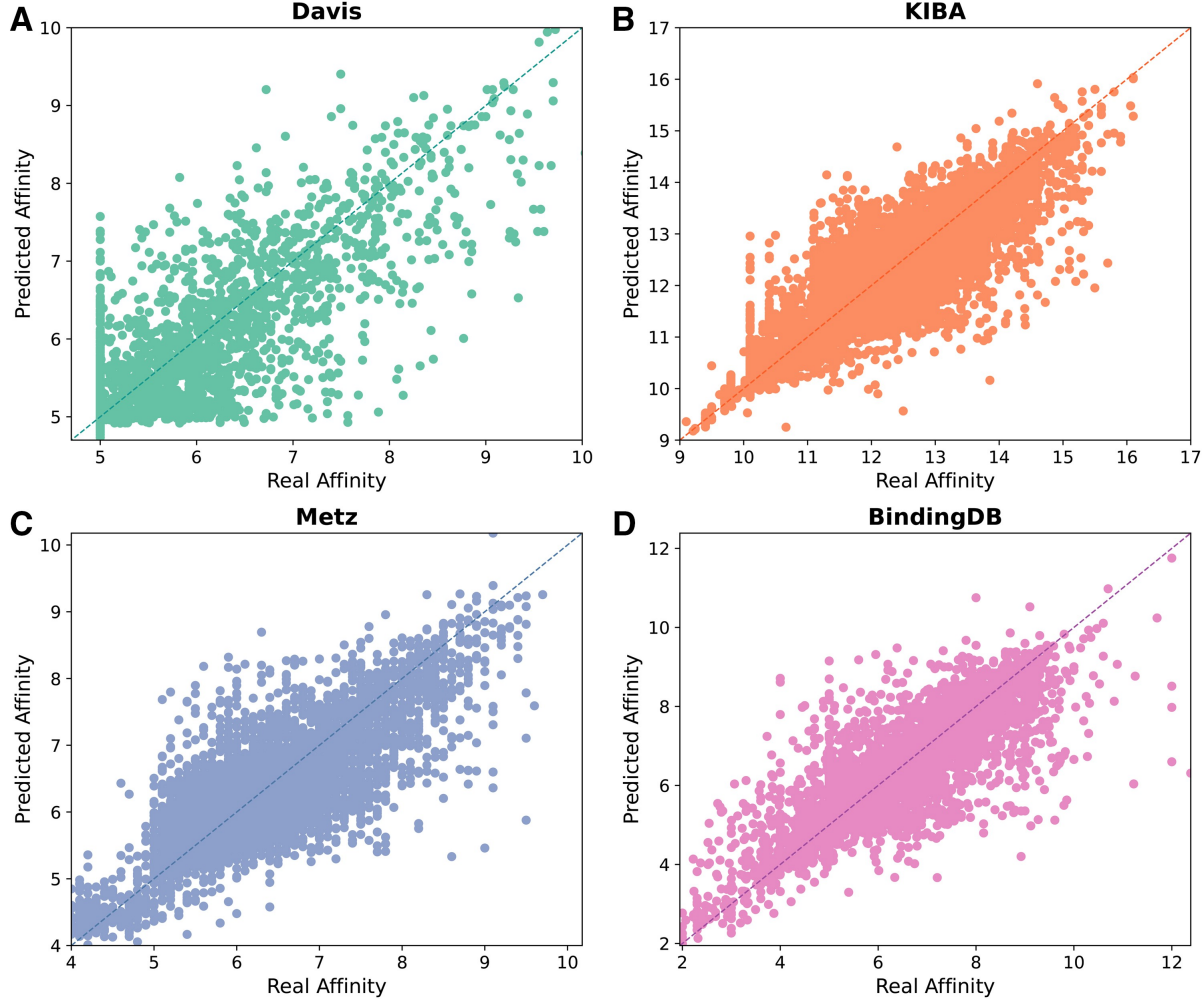
Comparison between predicted and real binding affinity values for TSEDTA on Davis (A), KIBA (B), Metz (C), and BindingDB (D) datasets.

To further validate the predictive ability of TSEDTA, we compared the predicted binding affinities with the true values across the four datasets, as illustrated in Fig. 3. Each point corresponds to a drug-target pair. A well-performing model produces points that are close to the diagonal line (*y* = *x*), indicating strong consistency between predictions and true values. As observed in the Fig. 3, most points are densely distributed around the *y* = *x* line. This result further confirms the robustness and reliability of TSEDTA in predicting DTA.

### 3.3 Ablation study

To investigate the contribution of the pretrained LLMs in encoding the drug and protein sequences, we designed a series of ablation experiments by removing the LLM components from the full model. TSEDTA incorporates a pretrained SMILES language model (SMILES Transformer) for drug representation and a pre-trained protein language model (ESM2) for protein embedding, both of which provide rich features. Once extracted, these features are mapped to the appropriate model dimensions through the projection layer. The processed features are then concatenated with the original embeddings, thereby enabling the integration of the pretrained LLMs. To evaluate the effect of the fusion process, we used the following ablation variants:

- w/o both-LLMs: Remove both SMILES Transformers for drug representations and ESM2 for protein representations.
- w/o SMILES Transformer: Remove SMILES Transformer for drug representations.
- w/o ESM2: Remove ESM2 for protein representations.

On the Davis dataset, the full TSEDTA model consistently demonstrates superior performance compared to its ablation variants. Specifically, when compared to the baseline variant lacking both LLM embeddings, TSEDTA improves the CI by 0.83%, reduces the MSE by 7.59%, and enhances the *r_m_*^2^ index by 3.49%. The exclusion of the SMILES Transformer leads to a 0.93% decrease in CI, a 4.77% increase in MSE and a 2.21% decrease in *r_m_*^2^. Notably, removing ESM2 results in the most significant performance degradation, with MSE increasing by 12.37%, CI and *r_m_*^2^ decreasing by 1.63% and 8.52% respectively, underscoring the critical role of evolutionary protein representations.

**Figure 4:**
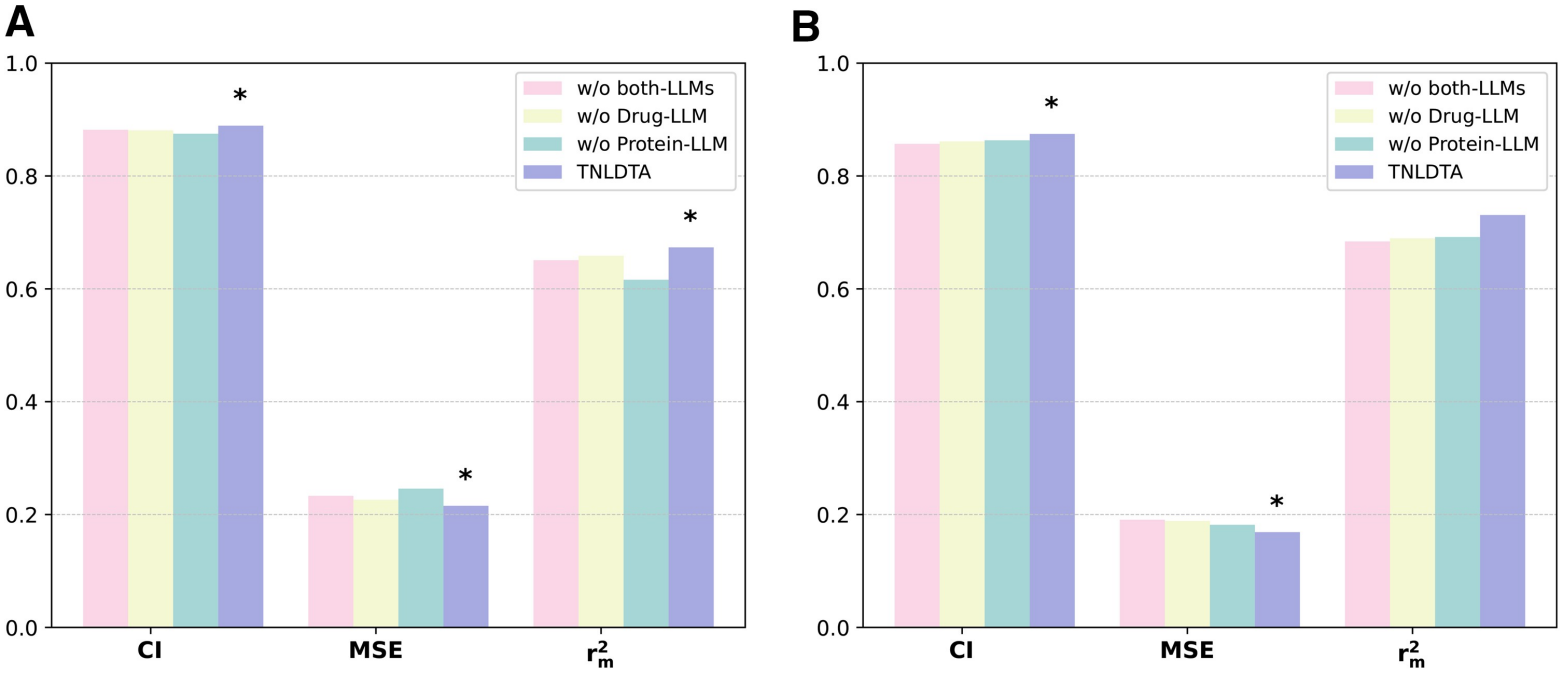
Ablation study results of TSEDTA on the Davis (A) and KIBA (B) datasets.

These improvements are even more pronounced on the KIBA dataset. Compared to the model without LLM integration, TSEDTA achieves a 2.05% and 6.84% improvement in CI and *r_m_*^2^ respectively, while simultaneously reducing the MSE by 11.53%. Compared to the variant without SMILES Transformer, the full model enhances CI, MSE, and *r_m_*^2^ by 1.54%, 10.55%, and 5.97%, respectively. Similarly, it surpasses the variant without the ESM2 by 1.31%, 7.15%, and 2.70% across these three metrics (Fig. 4, Table 5). These improvements across all metrics validated that incorporating pretrained LLMs effectively enhances the predictive capacity of TSEDTA for DTA prediction.

**Table 5:** Ablation experiment results of TSEDTA on Davis and KIBA datasets.

Dataset	Method	CI	MSE	$r_{m}^{2}$
Davis	w/o both-LLMs	0.8818	0.2331	0.6508
	w/o SMILES Transformer	0.8809	0.2262	0.6586
	w/o ESM2	0.8748	0.2458	0.6161
	TSEDTA	0.8891	0.2154	0.6735
KIBA	w/o both-LLMs	0.8569	0.1908	0.684
	w/o SMILES Transformer	0.8612	0.1887	0.6896
	w/o ESM2	0.8632	0.1818	0.6918
	TSEDTA	0.8745	0.1688	0.7308

### 3.4 Case study

While DTA prediction involves both proteins and ligands, the structural context of the protein is often a key determinant of its specificity. Therefore, we focused our interpretability analysis on the protein sequences to demonstrate that TSEDTA can effectively locate biologically active binding pockets without prior structural knowledge.

To evaluate the interpretability of TSEDTA, we selected two representative complexes (PDB IDs: 3AQV and 4ASD) for case studies. The model assigns attention scores at the residue level along the protein sequence, with higher scores indicating a stronger relevance to the predicted interaction. Residues corresponding to local maxima with attention scores exceeding 0.6 were considered potential interaction sites and spatially interpreted by mapping them onto the corresponding protein-ligand 3D structures. The ground truth interactions were defined as distances of less than 5.0 Angstrom.

**Figure 5:**
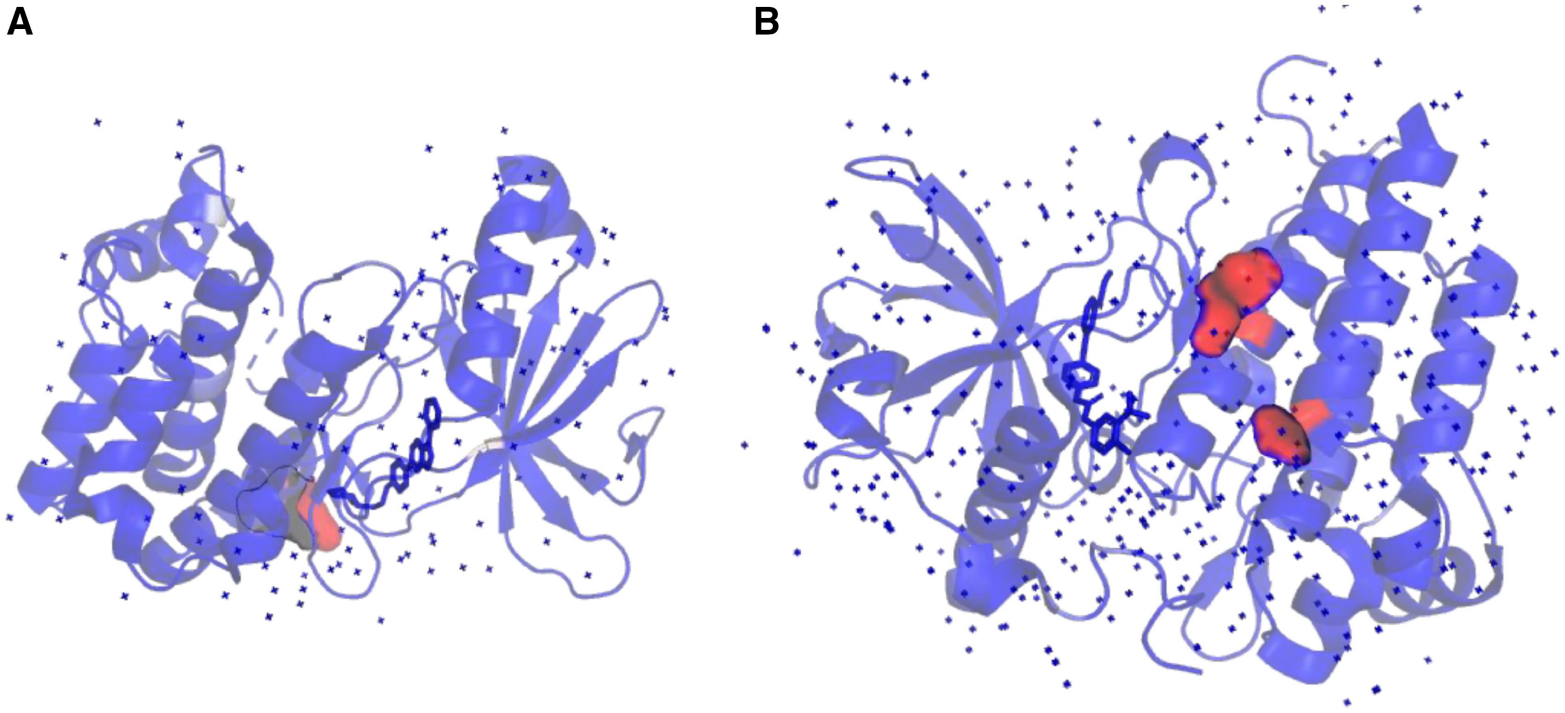
Structural visualization of protein–ligand interaction regions for two case studies. Red regions indicate residues associated with high attention scores assigned by TSEDTA. (A) Human AMP-activated protein kinase alpha 2 subunit kinase domain (T172D) complexed with compound C (PDB ID: 3AQV), where residue 104 is identified with the highest attention score at the binding interface. (B) Crystal structure of VEGFR2 (juxtamembrane and kinase domains) in complex with SORAFENIB (BAY 43–9006) (PDB ID: 4ASD). The model highlights residue 919 and 1108.

In the 3AQV complex, the ligand-binding pocket is defined by 13 residues (PDB indices 43, 45, 94–99, 103–104, 146, 156, and 164). Our model successfully identified residue 104 as having the highest attention weight. Notably, this high-attention residue belongs to the set of experimentally validated binding sites and is in close spatial contact with the ligand (highlighted in red in Fig. 5A). This demonstrates that the model successfully pinpointed key binding anchors solely from sequence information.

For the 4ASD complex, which contains 21 binding residues (PDB indices 840, 848, 866, 868, 885, 889, 892, 899, 916–920, 922, 1019, 1026, 1035, and 1044–1047), the model highlighted residues 919 and 1108. Notably, residue 919 is confirmed as one of the authentic ligand-binding sites (Fig. 5B), validating the model’s accuracy. The other highly attended residue, 1108, is located in a region far from the binding pocket. This phenomenon is frequently observed in attention-based architectures and typically represents a long-range dependency or a global information aggregation node, established by the model to capture the overall structural context of the protein.

## 4 Discussion

This study presents TSEDTA, a novel architecture utilizing SMILES Transformer and ESM2 embeddings for drug-target binding affinity prediction. By integrating these pre-trained models, TSEDTA captures profound structural and functional information. It fuses raw sequence embeddings with these domain-specific representations to retain the original information. A dual transformer encoder is employed to effectively model both the local and global dependencies within the drug and target sequences. The performance of TSEDTA is superior to that of the ten advanced models on both the Davis and KIBA datasets. Furthermore, extended evaluations on BindingDB dataset demonstrate its competitive predictive ability, successfully outperforming seven established baseline methods. Ablation studies further confirm that the full architecture consistently exceeds the performance of its ablation variants across KIBA and Davis datasets, thereby validating the design and effectiveness of the proposed model. Overall, TSEDTA is a promising tool for DTA prediction.

While recent advancements have introduced sophisticated architectures, incorporating attention mechanisms and structural graphs, a critical comparison reveals their underlying limitations regarding generalizability. Methods relying primarily on graph topology or traditional representation learning often capture 2D molecular structures effectively but struggle to encode deep, long-range biochemical semantics. Furthermore, models that train complex attention modules or standard Transformers entirely from DTA datasets are inherently constrained by the limited size and diversity of the task-specific training data. Without incorporating broad, domain-wide prior knowledge, these models tend to overfit the observed training distribution and frequently fail to generalize to out-of-distribution scenarios, such as novel chemical scaffolds or unseen target families. TSEDTA addresses these critical bottlenecks by leveraging the pre-trained contextual knowledge embedded within SMILES Transformer and ESM2. By mapping raw sequences into a robust, pre-learned semantic space, TSEDTA significantly improves generalization when evaluating novel drug-target interactions.

Although TSEDTA demonstrates excellent predictive performance, it has some limitations that warrant future investigation. First, while the model generalizes well to BindingDB, we observed performance degradation on the highly sparse Metz dataset. This indicates that while our pre-trained embeddings capture broad semantics, handling extreme data imbalance and sparsity mechanisms remains challenging. Second, the current model relies exclusively on 1D raw sequence information. The lack of explicit 3D spatial conformations can restrict the model’s generalization capabilities in scenarios where precise physical docking mechanisms heavily dictate binding affinity. In future work, we will consider using multi-source heterogeneous data and multi-scale interactive modules to improve the robustness. Additionally, we plan to incorporate structure-aware models, such as AlphaFold3(Abramson *et al.* 2024) to further enhance the cross-modal representation learning. Finally, we will explore comprehensive fine-tuning strategies for the pretrained models to better align the embedding space for DTA prediction.

## Supplementary Material

btag298_Supplementary_Data

## Data Availability

The source code underlying this article is actively maintained and available on GitHub at https://github.com/SunXu24Math/TSEDTA. A persistent snapshot of the code and experiments presented in this manuscript has been published on Zenodo with the DOI: 10.5281/zenodo.19103249. The supplementary materials, including detailed lists of proteins and ligands for all evaluated datasets, are also provided as supporting information.
