# Supplementary material for: TSEDTA: a transformer-based neural network with SMILES transformer and ESM2 embeddings for drug-target binding affinity prediction
Source: Bioinformatics. 2026 May 9;42(5):btag298. doi: 10.1093/bioinformatics/btag298 (PMC13211980; doi:10.1093/bioinformatics/btag298)
Supplement: btag298_Supplementary_Data [file btag298_supplementary_data.docx]

### Tables for Experimental Details

**Table S1:** Statistics of four benchmark datasets.

| Dataset | Number of Drugs | Average SMILES length | Number of Proteins | Average protein length | Interaction pairs |
| --- | --- | --- | --- | --- | --- |
| Davis | 68 | 63 | 442 | 746 | 30,056 |
| KIBA | 2111 | 59 | 229 | 729 | 118,254 |
| Metz | 170 | 45 | 1423 | 731 | 35,259 |
| BindingDB | 9864 | 78 | 1088 | 622 | 42,201 |

**Table S2:** Performance comparison between TSEDTA and other models on Metz dataset.

| Method | CI | MSE | $r_{m}^{2}$ |
| --- | --- | --- | --- |
| DeepDTA | 0.703 | 0.353 | 0.537 |
| GraphDTA | 0.801 | 0.317 | 0.62 |
| modality-DTA | 0.794 | **0.281** | - |
| MambaTransDTA | **0.804** | 0.302 | **0.636** |
| **TSEDTA** | 0.7955 | 0.3346 | 0.571 |

Note: bold values indicate the best results in each column.

Following the journal’s best practices, the large-scale numerical datasets are permanently archived on Zenodo (DOI: [10.5281/zenodo.19103249](https://doi.org/10.5281/zenodo.19103249)) for better accessibility and data integrity.

### 2. Model Architecture Details

**Position Encoding**

The positional encoding at each position pos and dimension j is defined as:

|  | $PE_{d}\left( k,2j \right)=sin\left( \frac{k}{{10000}^{\frac{2j}{d}}} \right), PE_{p}\left( k,2j+1 \right)=cos\left( \frac{k}{{10000}^{\frac{2j}{d}}} \right)$ | (S1) |
| --- | --- | --- |

**Multi-head Self-attention Mechanism**

Multi-head self-attention mechanism captures multiple types of dependencies in parallel, defined as:

|  | $P\text{MHAtt}\left( Q,K,V \right)=\sum_{i=1}^{h} \text{Attn}\left( QW_{i}^{Q},KW_{i}^{K},VW_{i}^{V} \right)W_{i}^{O}$ | (S2) |
| --- | --- | --- |
|  | $P\text{Attn}\left( Q,K,V \right)=\text{softmax}\left( \frac{QK^{\top}}{\sqrt{d_{k}}} \right)V$ | (S3) |

where W_i_^Q^, W_i_^K^, W_i_^V^, and W_i_^O^ are learnable weight matrices for query (Q), key (K), value (V), and output projections (O), and d_k_ is the dimension of the key vectors.

**Feed-Forward Network (FFN)**

The Feed-Forward network (FFN) processes each token independently, using a two-layer connected network:

|  | $\text{FFN}\left( x \right)=\text{ReLU}\left( xW_{1}+b_{1} \right)W_{2}+b_{2}$ | (S4) |
| --- | --- | --- |

**Residual Connection and Layer Normalization**

Residual connection facilitates training deeper networks by reducing gradient vanishing issues. And layer normalization stabilizes and accelerates training by normalizing activations across the feature dimension. They are defined as follows:

|  | $x_{\text{res}}=x+\text{Sublayer}\left( x \right)$ | (S5) |
| --- | --- | --- |
|  | $\text{LayerNorm}\left( x_{res} \right)=\frac{x_{res}-\mu}{\sqrt{\sigma^{2}+\epsilon}}$ | (S6) |

where μ and σ^2^ are the mean and variance across the feature dimension, and Sublayer (·) denotes the multi-head attention or feed-forward block.
